# Ageostrophic Frontal Processes Controlling Phytoplankton Production in the Catalano-Balearic Sea (Western Mediterranean)

**DOI:** 10.1371/journal.pone.0129045

**Published:** 2015-06-11

**Authors:** Temel Oguz, Diego Macias, Joaquin Tintore

**Affiliations:** 1 SOCIB, Balearic Islands Coastal Ocean Observing and Forecasting System, Parc Bit, Norte, Bloc A, Palma de Mallorca, Spain; 2 Middle East Technical University, Institute of Marine Sciences, 33731 Erdemli, Mersin, Turkey; 3 European Commission, Joint Research Centre, Institute for Environment and Sustainability, Water Resources Unit, Via E. Fermi, 2749, Ispra, Italy; 4 IMEDEA (CSIC-UIB), Miquel Marquès 21, 07190 Esporles, Spain; University of Vigo, SPAIN

## Abstract

Buoyancy-induced unstable boundary currents and the accompanying retrograde density fronts are often the sites of pronounced mesoscale activity, ageostrophic frontal processes, and associated high biological production in marginal seas. Biophysical model simulations of the Catalano-Balearic Sea (Western Mediterranean) illustrated that the unstable and nonlinear southward frontal boundary current along the Spanish coast resulted in a strain-driven frontogenesis mechanism. High upwelling velocities of up to 80 m d^-1^ injected nutrients into the photic layer and promoted enhanced production on the less dense, onshore side of the front characterized by negative relative vorticity. Additional down-front wind stress and heat flux (cooling) intensified boundary current instabilities and thus ageostrophic cross-frontal circulation and augmented production. Specifically, entrainment of nutrients by relatively strong buoyancy-induced vertical mixing gave rise to a more widespread phytoplankton biomass distribution within the onshore side of the front. Mesoscale cyclonic eddies contributed to production through an eddy pumping mechanism, but it was less effective and more limited regionally than the frontal processes. The model was configured for the Catalano-Balearic Sea, but the mechanisms and model findings apply to other marginal seas with similar unstable frontal boundary current systems.

## Introduction

**Fig 1:**
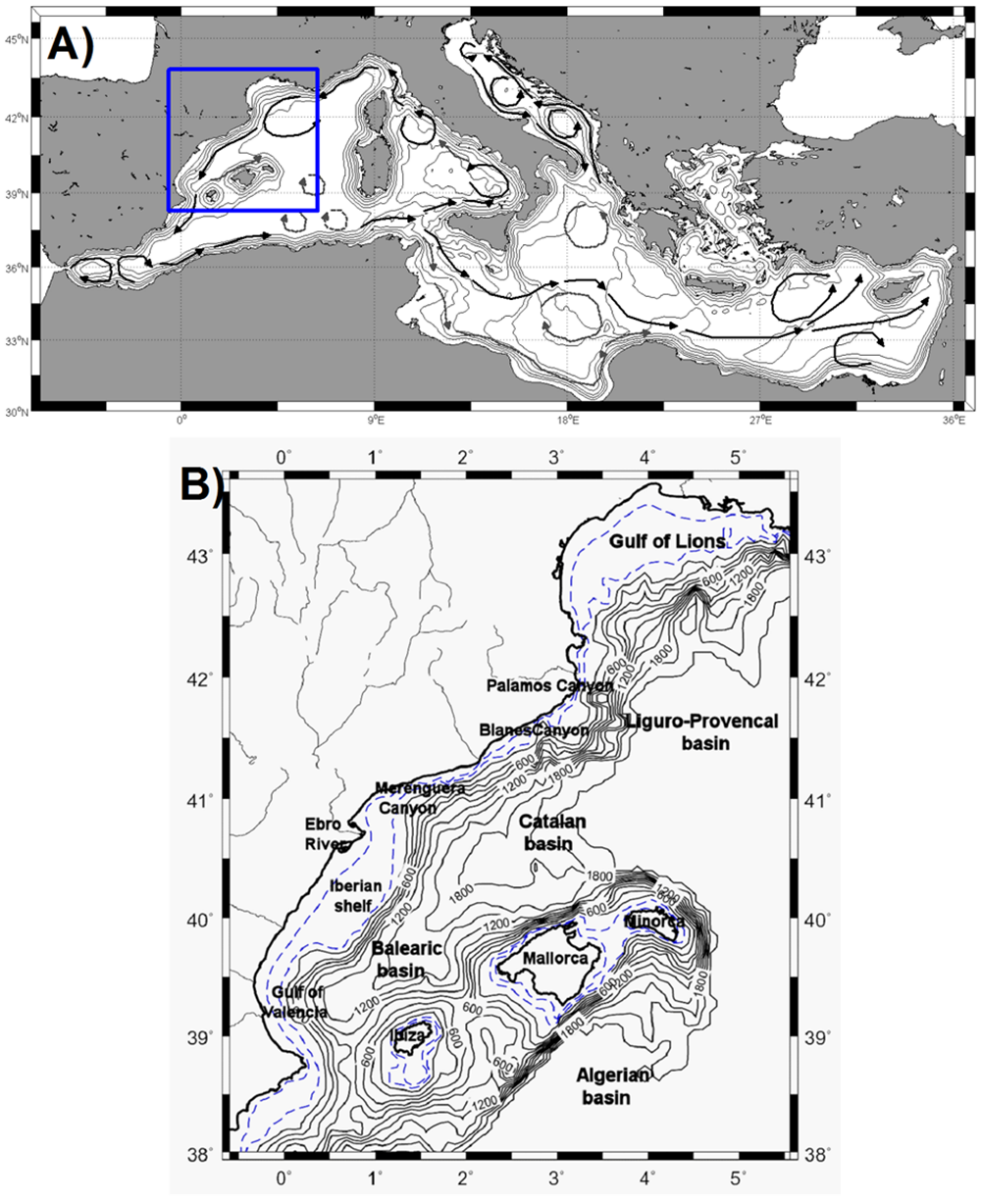
The model domain together with the smoothed bathymetry (depths in meters) used in the simulations as well as major morphological features referred to in the text. Dash lines represent 50 and 100 m isobaths.

The boundary current along the coast of the Catalano-Balearic (CB) Sea (Fig 1) constitutes a part of the Western Mediterranean cyclonic circulation system. It flows southward along the topographic slope zone with a maximum speed of approximately 0.3 ms^-1^ within the upper 100 m layer along the main jet axis, decreasing gradually to 0.05 ms^-1^ at 250 m to result in a total downstream transport of approximately 1 Sv [1,2]. In winter, the CB current strengthens [3], and its total transport can increase up to about 2 Sv [4]. This is accompanied by a well-marked front separating the relatively cold and fresh shelf/slope water from the warm and salty water of the interior basin [4–6]. The main portion of the current exits southward from the Ibiza channel, and the remainder bifurcates cyclonically to flow northeastward as the Balearic Current along the northern part of the Balearic Islands. This latter portion also interacts with the warmer and fresher water masses of the Algerian basin through the channels between the Balearic Islands. For a schematic depiction of the mean circulation pattern, refer to [3].

Enhanced mesoscale variability in the CB current has been observed in the field [4,6–11], in high-resolution data from gliders and multi-sensor measurements [12–14] performed by SOCIB (the Balearic Islands Coastal Ocean Observing and Forecasting System) [15], and in satellite data analyses [3,16] as mesoscale vortices, meanders, and filaments. These observations indicate biological enhancement at the mesoscale that may be driven by eddy-induced horizontal transports of nutrients and biota in the vicinity of frontal regions and their vertical transport by eddy pumping. These data and strong upward vertical velocities on the order of 10 m day^-1^ indicate a strong impact on the frontal zone due to the ageostrophic cross-frontal circulation [17–20]. Field observations also show enhanced chlorophyll concentrations and the preferential accumulation of zooplankton, fish eggs, and larval abundances at the onshore side of the meandering boundary current along the Catalan Sea [5,21–25], as well as the spreading of the gelatinous ctenophore species *Mnemiopsis leidyi* along the CB coast and around the Balearic Islands [26].

**Fig 2:**
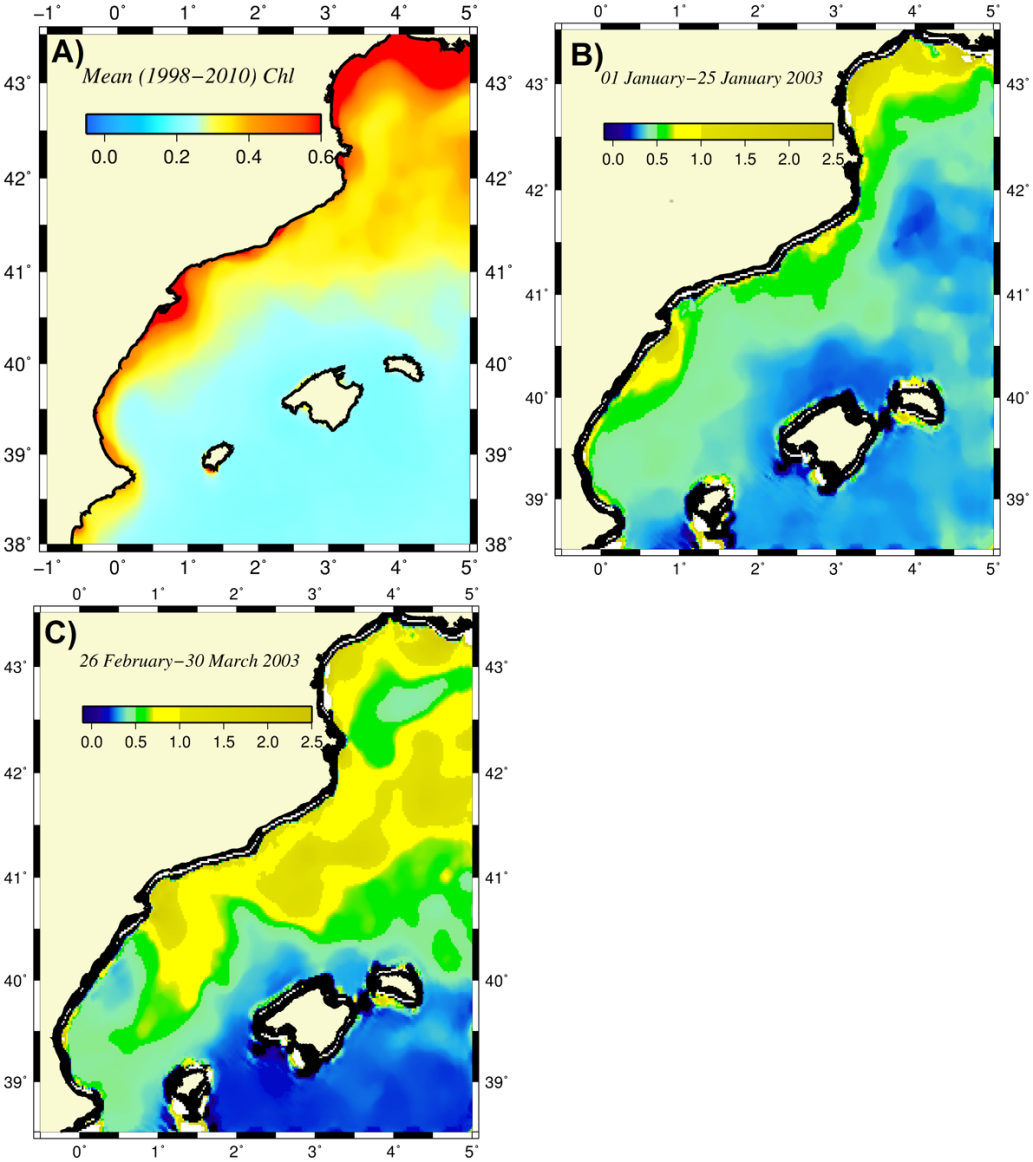
Distributions of the surface chlorophyll concentration (mg m^-3^) (a) as the average of all monthly data for 1998–2010, (b) for January 2003, and (c) for March 2003. The latter two chlorophyll images are constructed by the 4 km resolution, eight-day composite MODIS-Aqua satellite sensor products provided at http://gdata1.sci.gsfc.nasa.gov/daac-bin/G3/. The former is based on the 9 km resolution, monthly composite SeaWiFS data.

In addition to field observations, the long-term (1998–2010) mean ocean color-derived surface chlorophyll (chl-a) concentration data (Fig 2a) also showed relatively high concentrations of approximately 0.4–0.5 mg m^-3^ within the onshore side of the meandering frontal boundary current of the CB Sea that were comparable to concentrations within the adjacent deep convection site of the Liguro-Provencal basin and twice as high as those of the Western Mediterranean interior basin (Fig 2a). Focusing on the intra-annual structure, boundary current instabilities and cooling-induced vertical mixing appear to control production in winter (December-February) along the CB coast (Fig 2b and 2c). The satellite ocean color data suggest unproductive conditions during rest of the year because they are unable to capture the well-defined subsurface high chlorophyll layer typically observed in field data [21].

As expected, different mechanisms contribute to the generation and distribution of biological properties in the CB Sea. To date, there is no quantitative explanation for the relative roles of these mechanisms. In the present study, the concept of frontogenesis was applied to a baroclinically unstable buoyancy-driven coastal boundary current to demonstrate its strong control on phytoplankton production. In a strongly nonlinear flow regime, along-front geostrophic balance no longer holds because of strong cross-frontal density gradients and velocity shears. Subsequently, baroclinic instability leads to meanders, which create regions of high strain and vorticity due to intensification of nonlinearity and disruption of geostrophic balance. Strain generates frontogenesis and associated ageostrophic cross-frontal circulation [27–29]. This mechanism gives rise to high upward vertical velocities within anticyclonic eddies on the onshore side of the front along the Ligurian boundary current of the Western Mediterranean [30]. In a related study, Oguz et al. [31] described frontogenesis-induced enhanced autotrophic production along the meandering path of the narrow (15–20 km wide) Atlantic jet around the western and eastern anticyclonic gyres in the Alboran Sea.

The link between frontogenesis mechanisms and plankton production is substantiated here by a coupled physical-biological model describing how an initially stable frontal boundary current changed structurally in response to growing frontal instabilities and how the phytoplankton biomass distribution responded to these changes (1) in the absence of any atmospheric forcing and (2) in the presence of down-front wind stress and buoyancy-induced cooling. In the next section, we provide an overview of the frontogenesis mechanism and ageostrophic cross-frontal circulation (also referred to as ageostrophic secondary circulation) for readers who are not familiar with this concept. We then present a description of the model, its main findings, a discussion of the results, and the main conclusions.

## Overview of the Frontogenesis Mechanism

Different aspects of the frontogenesis mechanisms in open ocean frontal systems have been studied quantitatively [27, 28, 32–39]. Strong upwelling-downwelling motions along the frontal zone arise when the meandering of the baroclinically unstable front sharpens the cross-frontal density gradient because of the nonlinear interaction between the lateral velocity shear and buoyancy gradient. This process is expressed for the adiabatic case by [40]:

$$\begin{array}{l} \frac{d}{dt}{\left|\nabla_{h}^{2}\rho \right|}^{2}=Q\cdot \nabla_{h}^{}\rho \\ whereQ=-\left[\left(u_{x}\rho_{x}+v_{x}\rho_{y}),(u_{y}\rho_{x}+v_{y}\rho_{y}\right)\right] \end{array}$$ (1)

which states that the rate of change of the horizontal density gradient arises from its straining by the horizontal velocity field [28]. In Eq 1, ∇_*h*_ denotes the horizontal gradient operator, $\nabla_{h}^{2}$ the Laplacian operator, $\frac{d}{dt}$ the material time derivative. In Eq 1, *ρ* denotes the water density, and *u* and *v* represent the horizontal velocity components in the zonal and meridional directions, respectively, with the subscripts x and y representing the derivatives in the corresponding directions.

**Fig 3:**
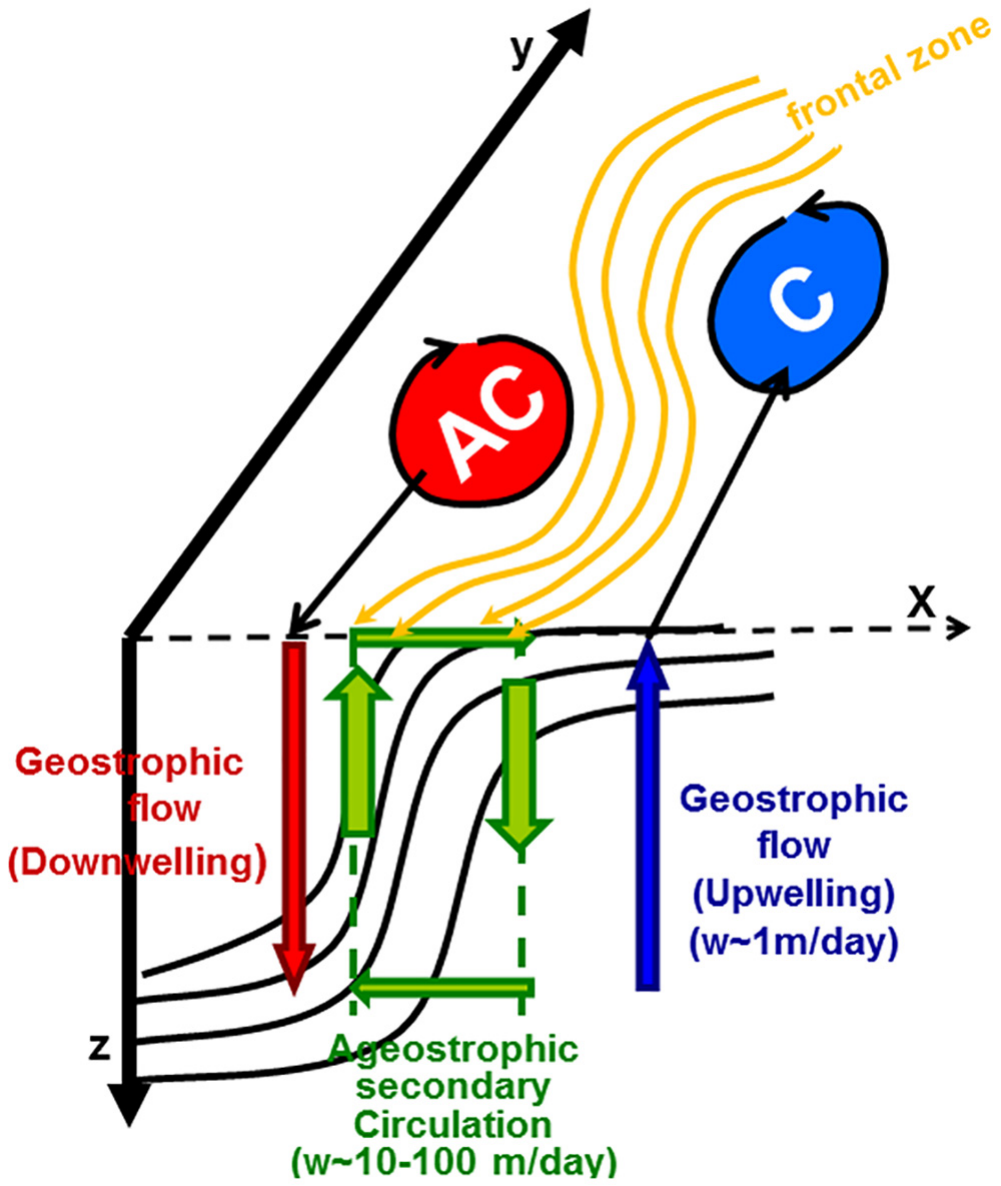
Schematic representation of the meandering boundary current and its accompanying anticyclonic and cyclonic mesoscale eddies as well as ageostrophic cross-frontal circulation with upwelling (downwelling) on the onshore (offshore) side of the frontal zone accompanied by quasigeostrophic dynamics prevailing further away from the frontal zone.

The consequence of the sharpening of fronts is the generation of locally narrow regions in which the lateral shear, strain rate, and relative vorticity become large and cause collapse of the along-front geostrophic balance. To re-establish the thermal wind balance of the along-front current, a cross-frontal vertical circulation cell is developed with intense vertical velocities on both sides. The vertical motion is directed downward in the troughs (i.e., onshore excursions of the frontal zone toward the less dense side), and their upstream side is characterized by cyclonic horizontal circulation. Conversely, upward vertical motion takes place in the crests (i.e., offshore excursion of the frontal zone towards the more dense side) and their upstream sides that acquire anticyclonic horizontal circulation. The cross-frontal velocity is convergent (i.e., from the anticyclonic to cyclonic side of the front) at the surface and divergent (i.e., from the cyclonic to anticyclonic side) at deeper levels. This vertical motion follows the conservation of potential vorticity in which a water column whose absolute vorticity decreases moving from a cyclonic to an anticyclonic region needs to be vertically compressed by upward vertical motion. Conversely, a water column whose absolute vorticity increases when moving from an anticyclonic to a cyclonic region needs to be vertically stretched by downward vertical motion. In other words, an upward motion characterizes regions that meander from troughs to crests, and those that meander from crests to troughs are characterized by a downwelling motion. A schematic representation of the meandering flow with quasi-geostrophic cyclonic and anticyclonic eddies, along with the frontal structure and ageostrophic cross-frontal circulation is depicted in Fig 3.

## Model Description

### Main features of the physical and biological models

The biophysical model comprised a coupled system of the Princeton Ocean Circulation Model (POM) and a four-compartment Nitrogen-Phytoplankton-Zooplankton-Detritus (NPZD) type biological model. The POM is a free-surface, primitive-equations model based on the f-plane, Boussinesq and hydrostatic approximations [41]. The original model was modified by implementation of a fourth-order pressure gradient algorithm to reduce the internal pressure gradient error [42, 43] and a third-order accurate upstream tracer advection scheme for temperature, salinity, and biological state variables [44]. Vertical viscosity and diffusivity were estimated by the 2.5-level Mellor-Yamada turbulence energy model [45]. Minimum values of the vertical viscosity and diffusivity were set to 2x10^-5^ and 1x10^-5^ m^2^ s^-1^, respectively. The horizontal friction terms in the momentum equations were parameterized in the Laplacian form with the horizontal viscosity defined by the sum of a constant value of 30 m^2^ s^-1^ and a flow-dependent contribution provided by the Smagorinsky parameterization. The horizontal diffusivity was set to half of the horizontal viscosity. The external (depth-integrated) equations were solved using time steps of 6 seconds and the internal (baroclinic) mode equations, as well as the biological equations using 300 seconds.

The biological model is similar to that implemented for the Alboran Sea of the Western Mediterranean [31]. Four-compartment biological models have been widely used for process-oriented modeling studies because their relatively simple trophic structure can better monitor the role of physical processes. Their use in the present study is further justified because the winter condition is primarily dominated by diatoms and copepods growing under low light and temperature and relatively high nutrient conditions in the CB Sea [5]. The main features of the model and its parameter values have previously been documented [31], and only the details relevant to this study are described below.

The model was configured for the domain shown in Fig 1, which comprised the CB Sea, the Gulf of Lions (GoL) to the north (> 41.5°N), and the northern Algerian basin in the south (< 39°N). The eastern open boundary was located at 6.5°E longitude. It employed an eddy-resolving rectangular grid of 0.04° (~3150 m) in the zonal direction and 0.025° (~2775 m) in the meridional direction. This grid structure resolved mesoscale features well, but it was not sufficiently fine to capture sub-mesoscale features [34]. Our simulations are therefore only valid for mesoscale ageostrophic motions. The model grid had 29 non-uniformly spaced vertical levels, 15 of which were located within the upper 200 m to better resolve the vertical biophysical processes.

### Initial conditions

The circulation model was initially in a state of rest and specified two different winter water masses for the interior basin and the shelf-slope regions. The upper 200 m of the entire deep basin (> 800 m) was filled by "old Mediterranean Atlantic Water" with T_s_ = 12.85°C and S_s_ = 38.2 psu. The upper 200 m of the northern and western coastal regions that were shallower than 800 m were occupied by a slightly colder and fresher water mass (T_s_ = 11.5°C and S_s_ = 37.5 psu). The layer between 200–600 m depths of the entire basin represented a slightly warmer and more saline “Levantine Intermediate Layer” water mass (T_i_ = 13.25°C and S_i_ = 38.5 psu) with the Mediterranean deep water mass underneath (T_d_ = 12.95°C and S_d_ = 38.55 psu). This setting created a surface intensified retrograde front with a density difference of 0.30 kg m^-3^ at the surface that agreed with typical observed conditions. The phytoplankton and zooplankton biomass and detritus concentration were initially set to low values throughout the basin. The nitrate structure was also horizontally uniform with values less than 0.5 mmol N m^-3^ within the upper 100 m layer that increased to its observed deep-water values below 200 m depth by a steeper slope. This structure was chosen to minimize the role of initial nitrate resources on the subsequent development of the three-dimensional plankton structure. It assures that production was governed solely by the upwelling induced by the ageostrophic motion. The vertical structures of the physical fields were also initially uniform within the upper 200 m layer and were later stratified by ageostrophic processes and atmospheric forcing.

### Boundary conditions

The model was linked to the rest of the Western Mediterranean by the eastern and southern open boundaries. The inflow was applied across the topographic slope zone of the northeastern open boundary in the following form:

uin=−u0Sin(πyLy)e−z/zbwithy≤Ly (2)

where *y* denotes the horizontal axis pointing to the north, *z*_*b*_ is the e-folding depth set 500m, u_0_ = 0.5 m s^-1^ the maximum inflow current, L_y_ = 10 Δy the width of the inflow over the slope zone extending from depths slightly greater than 2000m to nearly 200m. The inflow structure was kept unchanged for all simulations throughout the integration period and provided a total transport of approximately 1 Sv. Salinity and temperature at the inflow points held constant at their initial values. The internal and external modes of the flow field, except at the inflow boundary points, were treated by Orlanski-type free radiation conditions. The values of temperature and salinity along the open boundaries were evaluated from the adjacent interior grid points during the outflow conditions and were set to their values prescribed initially during the inflow conditions. At the bottom, adiabatic boundary conditions were applied to temperature and salinity, and quadratic bottom friction was applied to the momentum flux. No nitrate, plankton or detritus fluxes were specified at the lateral, bottom and surface boundaries. Wind stress and buoyancy flux at the surface was or was not prescribed depending on the type of simulations performed as specified below.

### Design of model simulations

**Fig 4:**
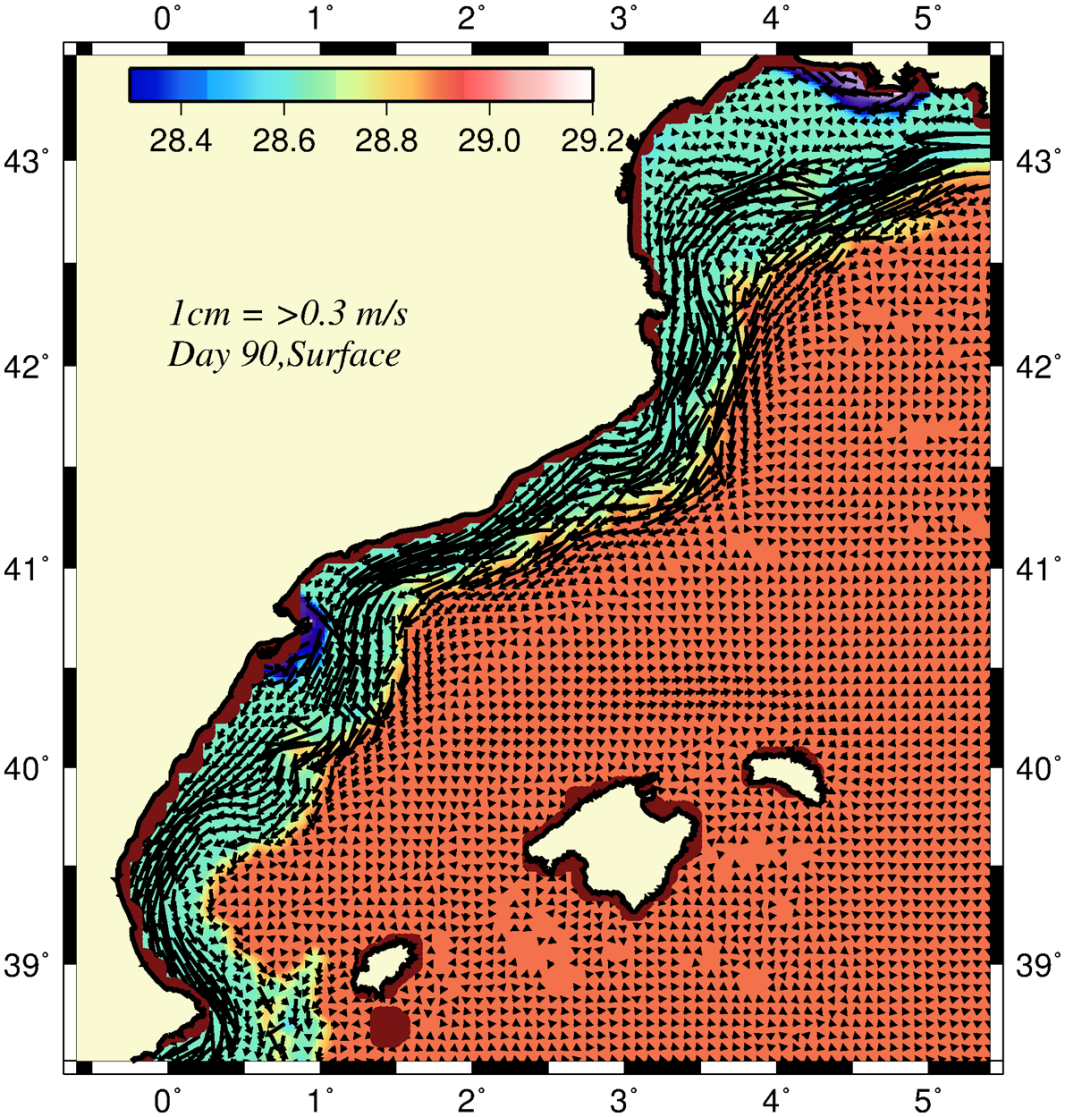
The surface flow field and density structure five days after the start of the model integration. They are considered as the geostrophically adjusted initial fields for the simulations.

For all simulations, starting from the initial conditions, the model was first integrated for 5 days to establish the geostrophically-adjusted frontal boundary current along the CB topographic slope (Fig 4). This solution was considered the initial flow state for rest of the integration period, and the model was integrated for another 90 days to monitor the nonlinear evolution of the baroclinically unstable frontal boundary current.

Four simulations were designed to explore different aspects of the ageostrophic frontal dynamics governing the plankton production characteristics of the CB region. The first considered the unforced frontogenesis mechanism, and the second and third extended the first to cases of weak atmospheric forcing from down-front wind stress (0.03 N m^-2^; northeasterly wind forcing) and buoyancy-induced cooling (rate = 50 W m^-2^), respectively. These simulations were representative of the mean winter conditions and therefore excluded any impacts of transient cold air outbreaks. The fourth simulation examined the impact of different zooplankton grazing rates. The surface wind stress was obtained from the bulk formula: τ_h_ = ρ_air_C_D_W^2^, where ρ_air_ = 1.3 kgm^−3^ is the density of air, C_D_ = 0.25*10^–3^ is the drag coefficient, and W = 3 ms^−1^ is the mean wind speed. When it was directed southwestward as in our model and the primary mode of the winter wind regime, the magnitudes of its zonal and meridional components were approximately 0.02 N m^−2^.

In the absence of any perturbation imposed on the unforced simulation, the boundary current structure remained stable without any signs of instability from coastal and topographic irregularities (i.e., natural perturbations) during its 90-day evolution. Therefore, for the unforced simulation, small-amplitude frontal perturbations were imposed within the upper 200 m of the salinity structure of the frontal zone between the 200 m and 1400 m isobaths to excite frontal instabilities. The default setting for the perturbations involved a single wave with period of 7 days, a wavelength of 50 grid sizes (roughly 150 km), and an amplitude of 1.0*10^–5^. It was applied as a single time step at the beginning of each day as a compromise between excessive dissipation and growth of unstable waves. This approach agreed with open ocean frontal models in which the initial geostrophically balanced jets were perturbed to excite instabilities.

An extensive set of experiments explored the sensitivity of the model to the initial cross-frontal density contrast, perturbation characteristics (e.g., amplitude, period and wavelength), and biological parameters. However, a description of their details are beyond the scope of the present work. The simulations described below also switched off the river fluxes to exclude their contribution to the development of the frontal structure. This issue will be the subject of a separate study. The values of the biological parameters chosen for this work were similar to those of our previous modeling study devoted to the region [46].

## Development of Ageostrophic Frontal Motion and Plankton Production

### Adiabatic case in the absence of atmospheric forcing

**Fig 5:**
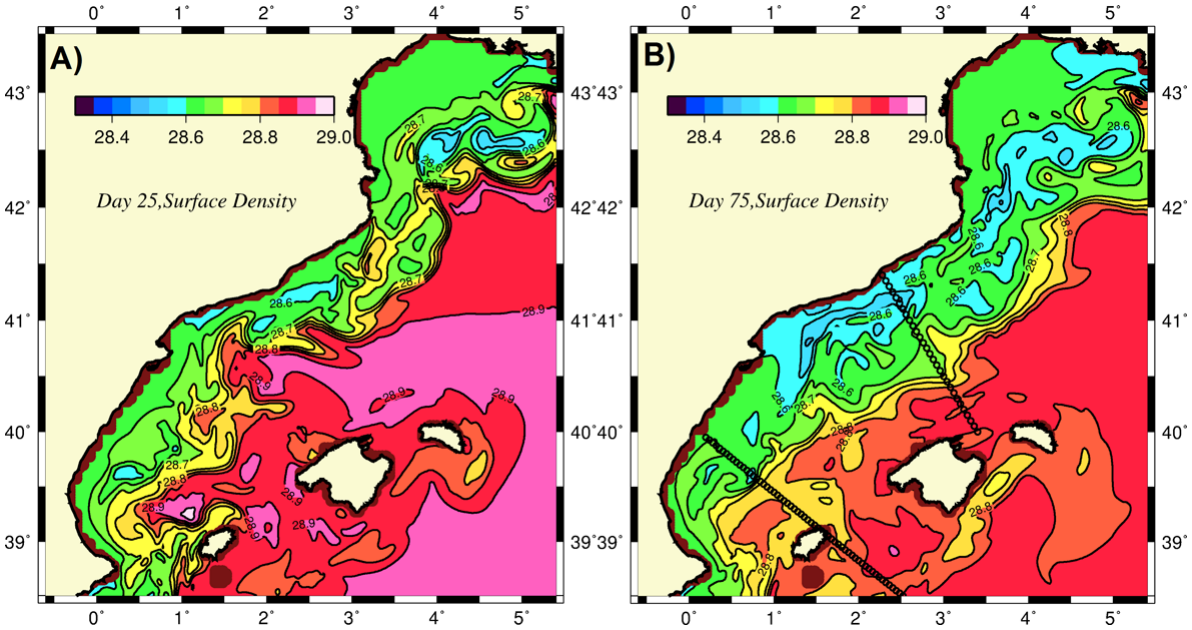
Surface distributions of density at (a) day 25 and (b) day 75 for the abiabatic (unforced) simulation. The dotted lines represents the transects used to represent cross-sectional variations along the Catalan and Balearic Seas.

For the unforced frontogenesis case, once the initial stable front-jet structure was perturbed, instabilities quickly developed and deformed the frontal structure depending on the amplitude of the perturbations. For small amplitude perturbations (the default case), frontal instabilities were rather weak. Nevertheless, the front that initially conformed along the upper topographic slope was displaced to approximately 100 km offshore and started producing meanders. In the early days of flow development (Fig 5a), the onshore side of the front was homogenized with relatively uniform low-density waters approximately 28.65±0.05 kg m^-3^, whereas the front itself displayed steep density changes of approximately 0.2 kg m^-3^ at a distance less than 10 km. Once the unstable flow structure evolved to its fully nonlinear regime, the front expanded further offshore, and its density contrast was slightly weakened. The water mass on its onshore side also became slightly less dense (28.6 kg m^-3^) because of the continuous fresh water supply from the GoL region (Fig 5b).

**Fig 6:**
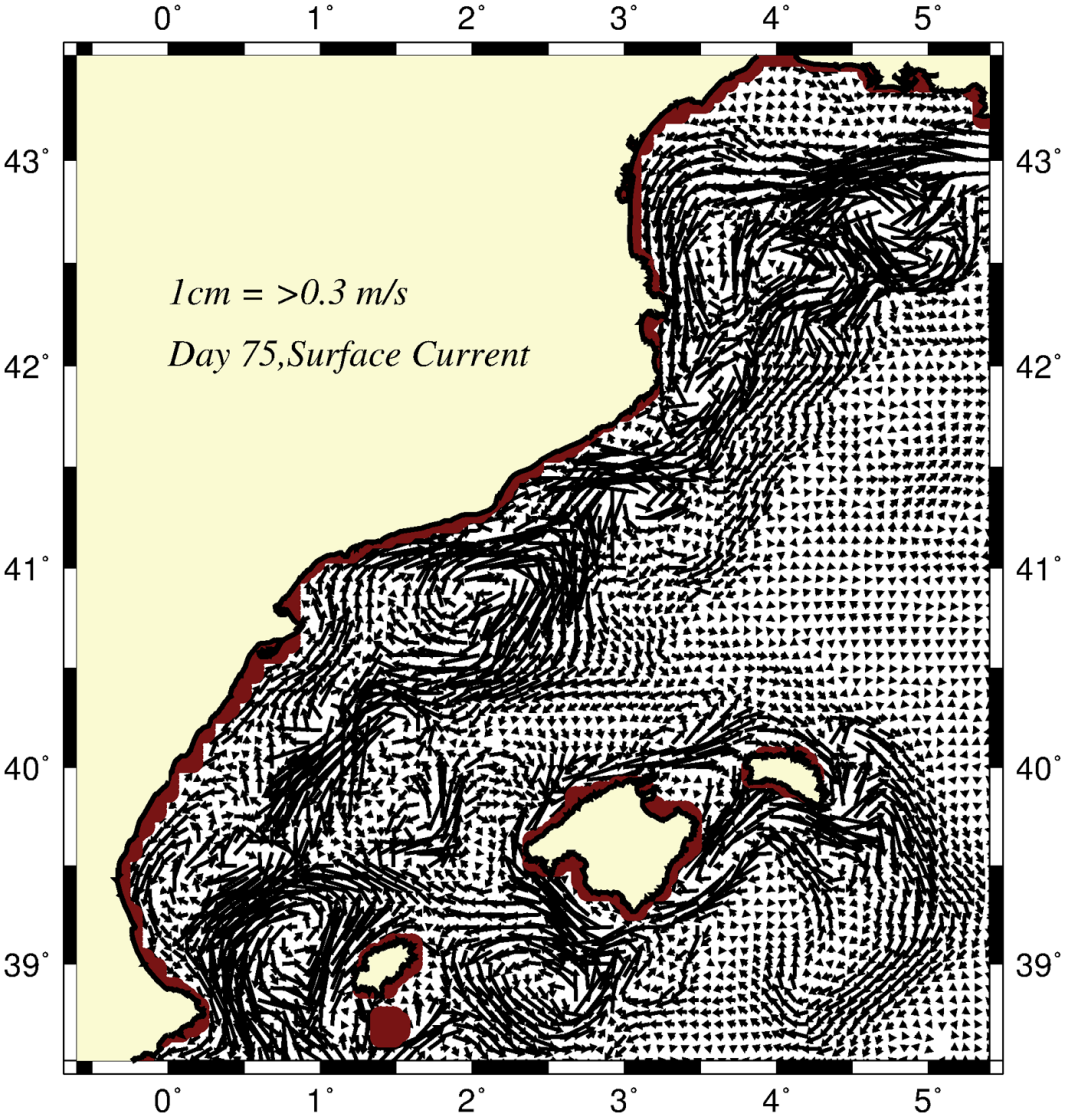
Horizontal flow field at day 75 for the abiabatic (unforced) simulation.

Inflow entering from the French coastal zone at the northeastern corner of the model domain crossed the GoL zonally along the shelf break and the upper slope topography in the form of two branches. These branches then formed the coastal-inner shelf current and the upper topographic slope current along the Spanish coast (Fig 6). The third branch of the inflow flowed southward along the margin topography of the Catalan basin coinciding with the CB density front. The shelf break-slope current, the major component of the CB boundary current structure, first meandered onshore, then offshore between the Blanes and Merenguera canyons in the Catalan Sea (see Fig 1). These meanders were associated with a cyclonic and anticyclonic eddy pair located offshore and onshore side of the boundary current, respectively. The anticyclonic eddy occupied the Merenguera canyon region.

Along the Iberian shelf break, the slope current was accompanied by an eddy pair as well: an anticyclonic eddy inside the Iberian shelf and a cyclonic eddy within the deep Balearic basin (Fig 6). The offshore branch of the cyclonic eddy also supported a relatively weak eastward current (the so-called Balearic Current) over the topographic slope of the northern side of the Mallorca and Minorca Islands. It then circulated around the Balearic Islands and produced northward protrusions across the Mallorca and Ibiza channels. The latter was deflected cyclonically within the Balearic basin and exited from the western side of the Ibiza channel together with the CB boundary current. The Balearic basin was more populated by mesoscale eddies.

**Fig 7:**
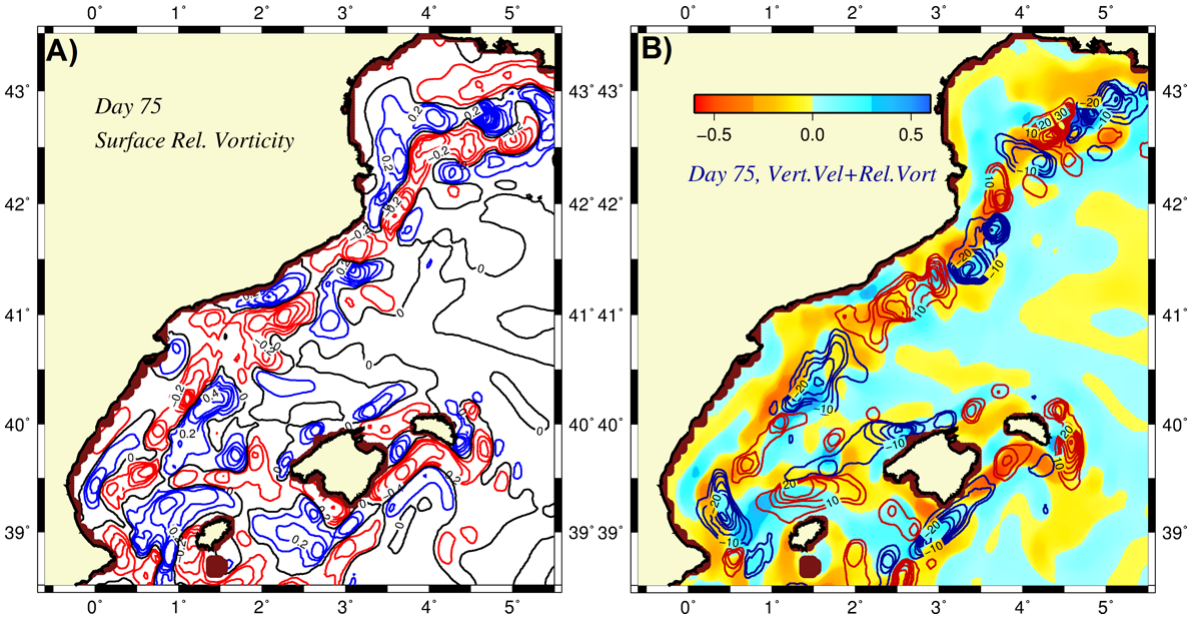
Horizontal distributions of (a) the surface relative vorticity (normalized by the planetary vorticity) with the contours in red (blue) color representing anticyclonic (cyclonic) vorticity, (b) the vertical velocity (m day^-1^) with the contours in red(blue) color representing upward(downward) motion at day 75 of the adiabatic experiment. The background color in the vertical velocity distribution represents the regions of anticyclonic (cyclonic) vorticity in yellow(blue) color.

**Fig 8:**
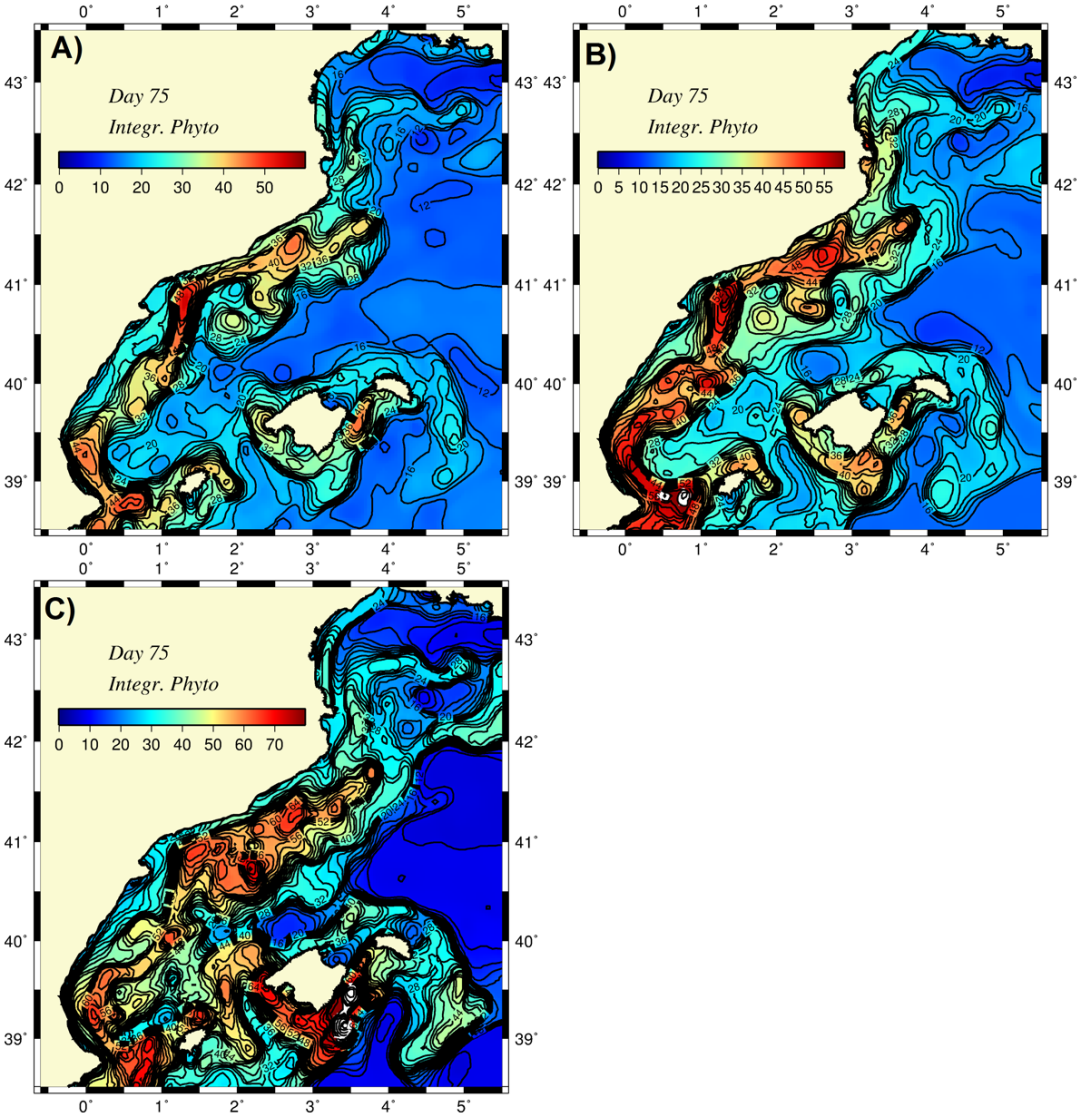
Distributions of depth integrated (over 100 m) phytoplankton biomass at day 75 for (a) the abiabatic (unforced) simulation, and diabatic case with (b) down-front wind forcing, (c) buoyancy-induced cooling.

Major features of the flow field were also depicted by the surface relative vorticity distribution (normalized by the planetary vorticity) in Fig 7a. The meandering boundary current was characterized by a narrow vorticity gradient zone between its negative values on the anticyclonic onshore side and positive values on the cyclonic offshore side of the front. The magnitudes of some of these cyclonic and anticyclonic eddies were approximately 0.5, indicating a moderately nonlinear flow field. As shown by the vertical velocity distribution at 50 m (Fig 7b), the boundary current frontal region involved a sequence of upwelling-downwelling centers where the regions of upward motion coincided with the regions from troughs to crests and vice versa for the downward motion. Thus, anticyclones located on the onshore side of the meandering frontal boundary current were generally associated with upwelling motions and acquired more efficient nutrient injections from the subsurface levels. On the other hand, the cyclonic eddies located on the offshore side of the boundary current experienced downwelling motions and thus drained nutrients from the photic zone to deeper depths. These features are consistent with the frontogenesis mechanism and the related ageostrophic cross frontal circulation. As inferred by the depth-integrated phytoplankton biomass distribution in Fig 8a, the anticyclonic coastal eddies emerged as preferential sites of phytoplankton biomass production and accumulation, whereas the cyclonically-dominated offshore side of the front remained deficient in biological production.

**Fig 9:**
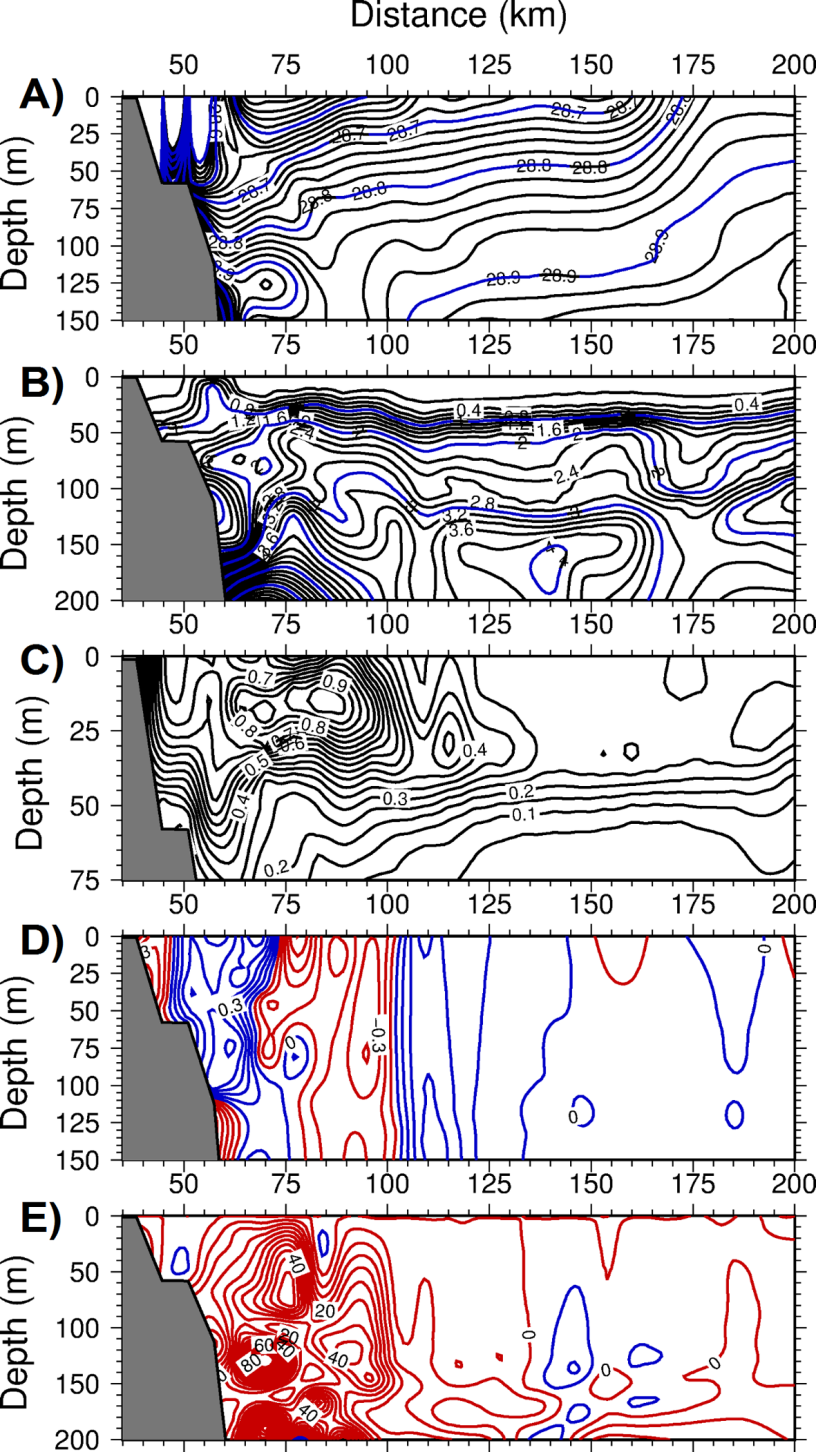
The depth versus offshore (cross-front) variations within the Catalan basin for (a) density (kg m^-3^), (b) the nitrate concentration (mmol m^-3^), (c) phytoplankton biomass (mmol m^-3^), (d) relative vorticity (normalized by the planetary vorticity) with the contours in red(blue) color representing anticyclonic (cyclonic) vorticity, (e) the vertical velocity (m day^-1^) with the contours in red(blue) color representing upward(downward) motion at day 75 of the adiabatic experiment.

**Fig 10:**
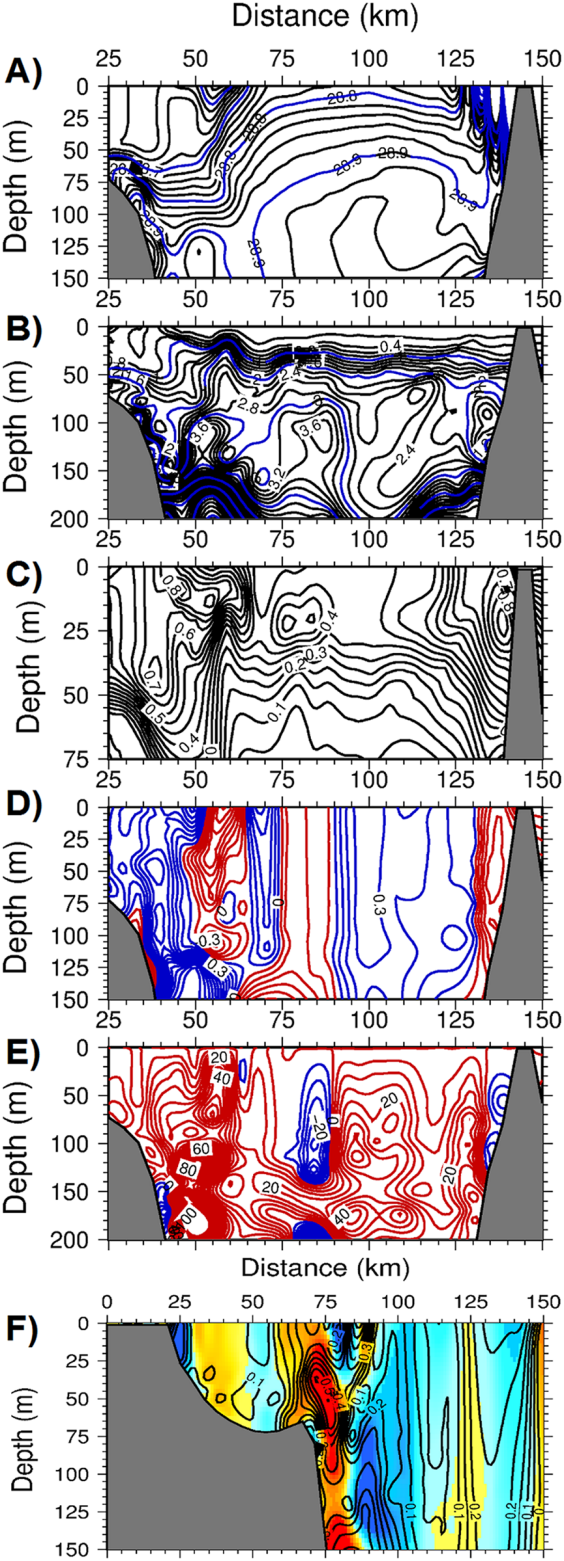
The depth versus offshore (cross-front) variations within the Balearic basin for (a) density (kg m^-3^), (b) the nitrate concentration (mmol m^-3^), (c) phytoplankton biomass (mmol m^-3^), (d) relative vorticity (normalized by the planetary vorticity) with the contours in red(blue) color representing anticyclonic (cyclonic) vorticity, (e) the vertical velocity (m day^-1^) with the contours in red(blue) color representing upward(downward) motion, (f) strain rate with the backgroung color representing the relative vorticity at day 75 of the adiabatic experiment.

Further details on the features of the frontogenesis mechanism were revealed along two offshore transects (see Fig 5b for their locations). The transect across the Catalan Sea off of Barcelona exhibited both the inner shelf and offshore fronts located 60 km and 100 km away from the coast, respectively (Fig 9a). The region in between these fronts coincided with the southward current and was characterized by anticyclonic (negative) relative vorticity down to -0.5 (Fig 9d) and upward vertical velocities up to 50 m d^-1^ (Fig 9e). These parameters introduced considerable cross- isopycnal nitrate transport in the subsurface (Fig 9b) and gave rise to phytoplankton biomass up to 1 mmol m^-3^ within the upper 50 m of the anticyclonic region (Fig 9c). Light limitation prevented this feature from extending to deeper depths. Similar features were also observed in the transect across the Balearic basin. The well-defined density front over the topographic slope zone off of the Iberian shelf (Fig 10a) was associated with an anticyclonic vorticity down to -0.8 (Fig 10d) and an upward vertical velocity up to 80 m d^-1^ (Fig 10e). These parameters supported a phytoplankton biomass of up to 0.8 mmol m^-3^ (Fig 10c). A similar frontal production process resulting from the ageostrophic dynamics occurred on the southeastern edge of the transect in the vicinity of the northern flank of Ibiza Island.

The frontal zones of both transects were associated with relatively high strain rates (i.e., horizontal velocity shears) arising from instabilities in the boundary current (Fig 10f). The strong horizontal shears combined with the similarly strong lateral density gradients across the front (Fig 5a and 5b) were key for the strength of the ageastrophic frontal motions. Moreover, subduction events taking place on the offshore side of the front were relatively weak, as with the slight downwelling of nitrate concentrations within a narrow zone on the offshore side of the fronts (Figs 9b and 10b).

As shown by the density transects in Figs 9a and 10a, the interiors of the Catalan and Balearic basins beyond the frontal zones were characterized by considerable stratification due to the prevailing upwelling motions (less than 5 m d^-1^) associated with cyclonic (positive) relative vorticity values of 0.1–0.3. They therefore reflected the characteristics of the quasi-geostrophic motion governing the overall flow field beyond the frontal zone. These regions were moderately productive and contained phytoplankton biomasses of 0.3–0.4 mmol m^-3^, with approximately 30% to 50% of those developed within the frontal regions.

### Diabatic case with down-front wind forcing

**Fig 11:**
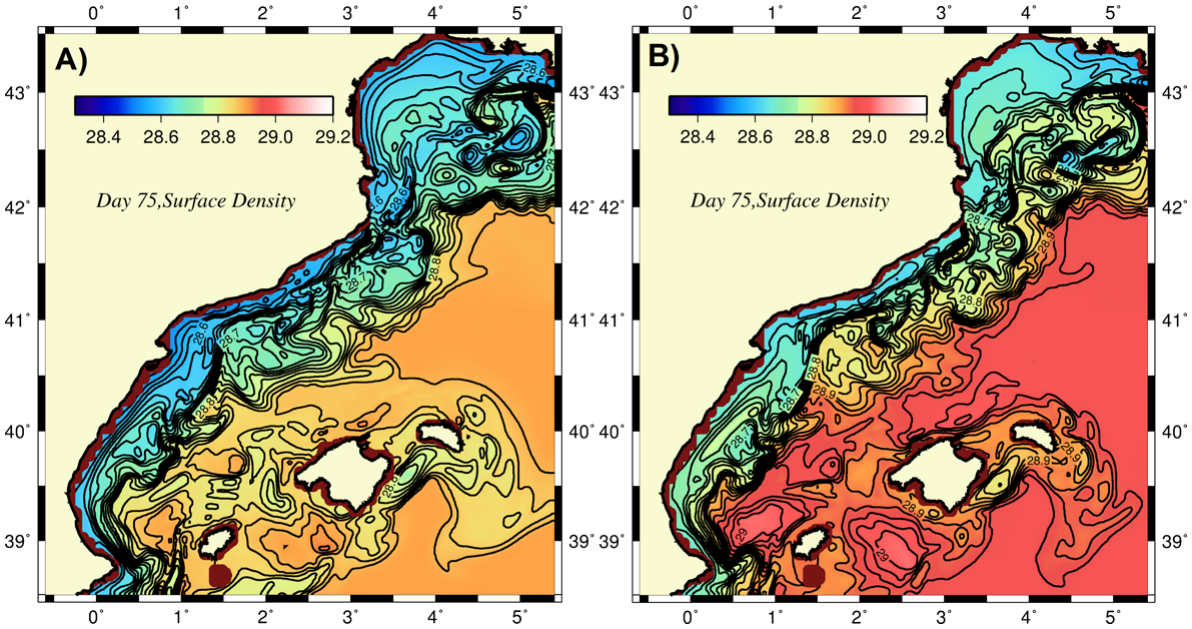
Distributions of surface density at day 75 for the diabatic simulations with (a) down-front wind forcing, (b) buoyancy-induced cooling.

The previous unforced simulation was extended to include a steady northeasterly wind forcing that was roughly oriented along the frontal jet (i.e., down-front). Its magnitude, 0.02 N m^-2^, in both the zonal and meridional directions represented mean conditions for the winter season and excluded relatively strong episodic cold air outbreaks. The resulting Ekman transport associated with this wind stress advected surface waters from the denser to the lighter side of the front and caused gravitational instability of the front and its subsequent erosion by the onshore protrusion of dense waters and the gradual migration of the front towards the lower density region (Fig 11a) [27, 39, 47]. Stronger vertical mixing increased the density of surface waters on the onshore side of the front by roughly 0.1 kg m^-3^ relative to the unforced case.

**Fig 12:**
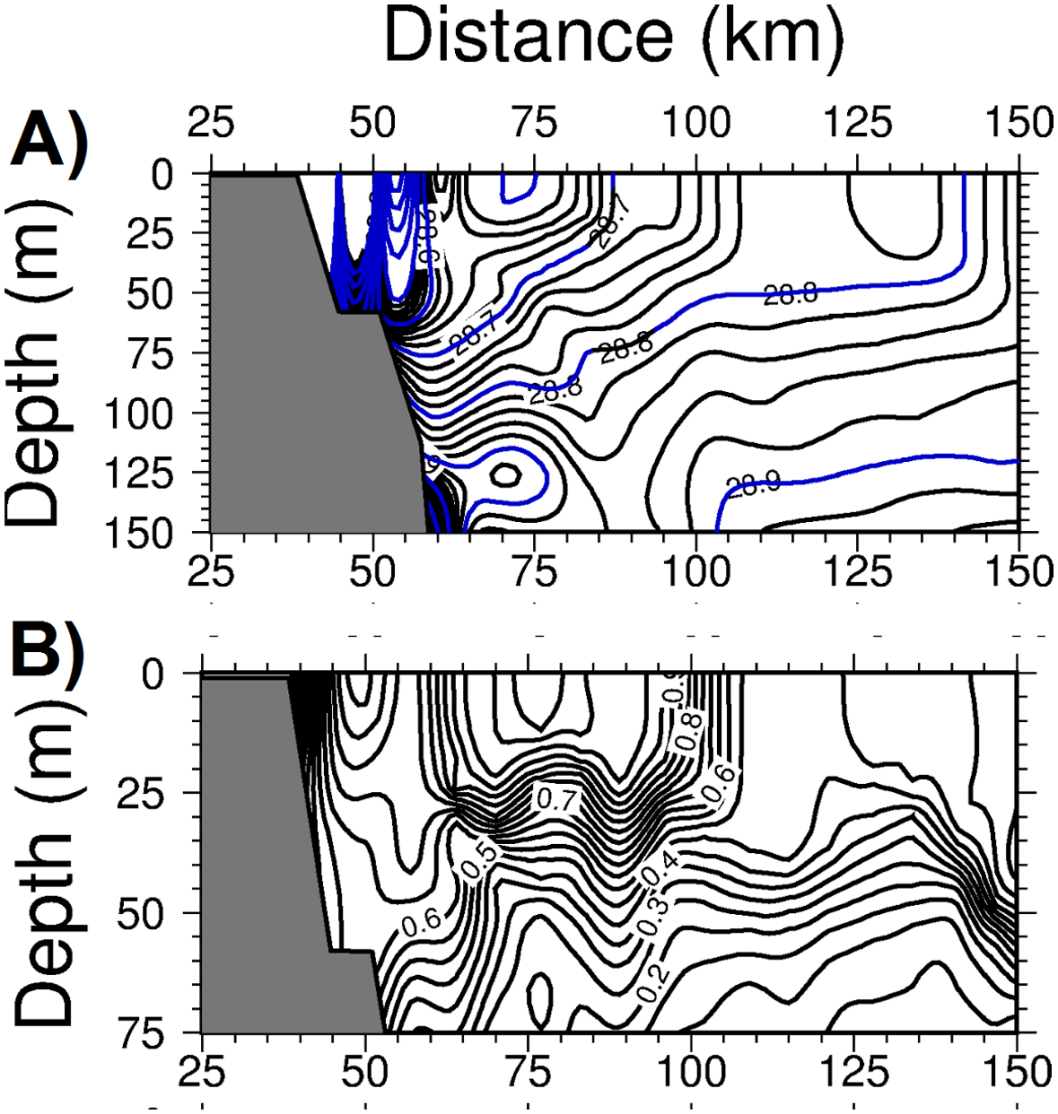
The depth versus offshore (cross-front) variations within the Catalan basin for (a) density (kg m^-3^), (b) phytoplankton biomass (mmol m^-3^) at day 75 of the diabatic experiment with down-front wind forcing.

The signature of the gravitational-induced vertical mixing was more clear in the density transect across the Catalan basin (Fig 12a). Relative to the unforced case, the upper 50 m of offshore waters acquired higher densities (~0.1 kg m^-3^) with the outcropping of isopycnals with a densities of 28.7–28.8 kg m^-3^ and a slightly narrower, but stronger frontal structure. A higher rate of nitrate entrainment by vertical mixing gave rise to a more vertically uniform and slightly higher phytoplankton biomass structure over 50 m depth within the frontal zone (Fig 12b). This feature was also supported by a slightly higher integrated phytoplankton biomass distribution within the frontal zone relative to the unforced case (Fig 8b).

### Diabatic case with buoyancy-induced cooling

The impact of surface buoyancy loss on frontal systems has not been well studied. Levy [35] noted a gradual deepening of the mixed layer and surface intensification of the plankton production with respect to cases in the absence of cooling. Nagai et al. [37] emphasized the critical role of enhanced vertical mixing. In the absence of this feature, their model did not reproduce any phytoplankton blooms on the lesser dense side of the front. In our case, prescribing a steady cooling rate of 50 W m^-2^ over the basin, representative of the mean winter cooling rate, triggered convection and led to stronger mixing of physical and biological properties within the upper layer of the water column. At the same time, weakening of the stratification within the frontal zone enhanced the baroclinic instability of the frontal boundary current (Fig 11b) and translated the frontal structure towards the coast, making the low-density region narrower with respect to the unforced case. The surface density increase due to cooling and entrainment in this region was approximately 0.2 kg m^-3^, whereas the corresponding increase in the interior basin surface waters was limited to 0.1 kg m^-3^. The surface relative vorticity field revealed stronger vorticity centers close to unity (not shown) and more pronounced vorticity gradients at frontal regions, where the density gradients were also stronger compared to the unforced case. Furthermore, while vertical mixing increased mesoscale variability within the frontal zone by means of baroclinic instability, it also smoothed out or weakened the mesoscale structures within the Balearic basin and around the Balearic Islands.

In addition to enhancing the baroclinic instability of the frontal boundary current, the loss of buoyancy also modified the stratification characteristics in the upper layer of the water column and introduced stronger entrainment mechanisms that brought more nutrients into the euphotic layer. This subsequently resulted in larger and more widespread phytoplankton production due to the distribution of nutrients by the mesoscale eddies and boundary currents (Fig 8c). A comparable entrainment rate was, however, not equally effective in bringing nutrients into the surface layer within the offshore waters beyond the frontal zone because of a lack of a strong upwelling motion in the ageostrophic motion. This implies that pure convection-induced biological production within the interior waters is only possible when strong deep convection events follow strong cold air outbreak events.

**Fig 13:**
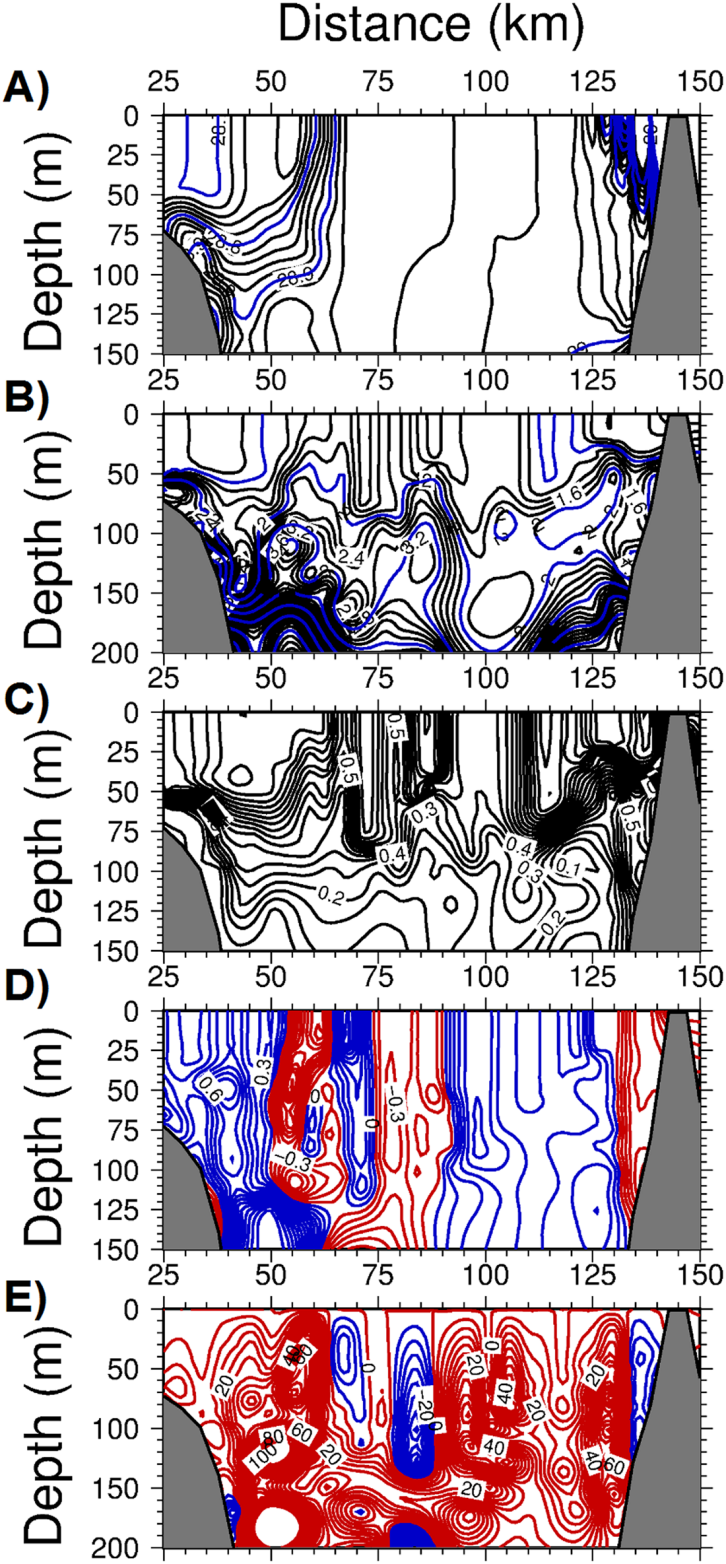
The depth versus offshore (cross-front) variations within the Balearic Sea for (a) density (kg m^-3^), (b) the nitrate concentration (mmol m^-3^), (c) phytoplankton biomass (mmol m^-3^), (d) relative vorticity (normalized by the planetary vorticity) with the contours in red(blue) color representing anticyclonic (cyclonic) vorticity, (e) the vertical velocity (m day^-1^) with the contours in red(blue) color representing upward(downward) motion, at day 75 of the diabatic experiment with buoyancy-induced cooling.

The considerable modifications to the vertical structure in the transect across the Balearic Sea are shown in Fig 13 with respect to the unforced case shown in Fig 10. The major alterations consisted of erosion of the stratification within the upper 100 m, outcropping of 28.8 and 28.9 kg m^-3^ isopycnals at the surface within the interior region between the two frontal zones on both sides of the transect, and a stronger cross-frontal density gradient on the western front between 28.75–28.95 kg m^-3^ (Fig 13a). Deepening of the surface mixed layer however did not take place within the frontal zone or its onshore side because of a strong upward motion counterbalancing the buoyancy-induced destratification. The front was accompanied by strong anticyclonic vorticity close to unity (Fig 13d), and upward vertical velocities up to 80 m d^-1^ (Fig 13e). The offshore side of the front was characterized by a cyclonic vorticity zone associated with a strong downwelling vertical motion (Figs 13d and 13e). Fig 13c shows relatively high phytoplankton biomass generation on the onshore side of the front and its subduction on the offshore side (extended to 85 km along the transect). The coastal region to the west of the front was identified by a cyclonic vorticity and upward vertical velocities of approximately 20 m d^-1^ that supported a phytoplankton biomass comparable to that of the frontal zone. Two similar narrow zones on both sides of the location at 100 km along the transect had similar features. They were characterized by a relatively weak cyclonic vorticity (< 0.5), but relatively strong upward vertical velocities of up to 40 m d^-1^ and high phytoplankton biomass zones. A clear upwelling signature was evident from the upward rising of the nitraclines in these two narrow upwelling zones (Fig 13b). A similar signature of upward rising nitraclines was also noted at the frontal zone. Thus, the Balearic transect had contributions from frontal processes, but also the quasigeostrophic eddy pumping and related mesoscale processes to the biological production in the CB region.

### Higher zooplankton grazing rates

**Fig 14:**
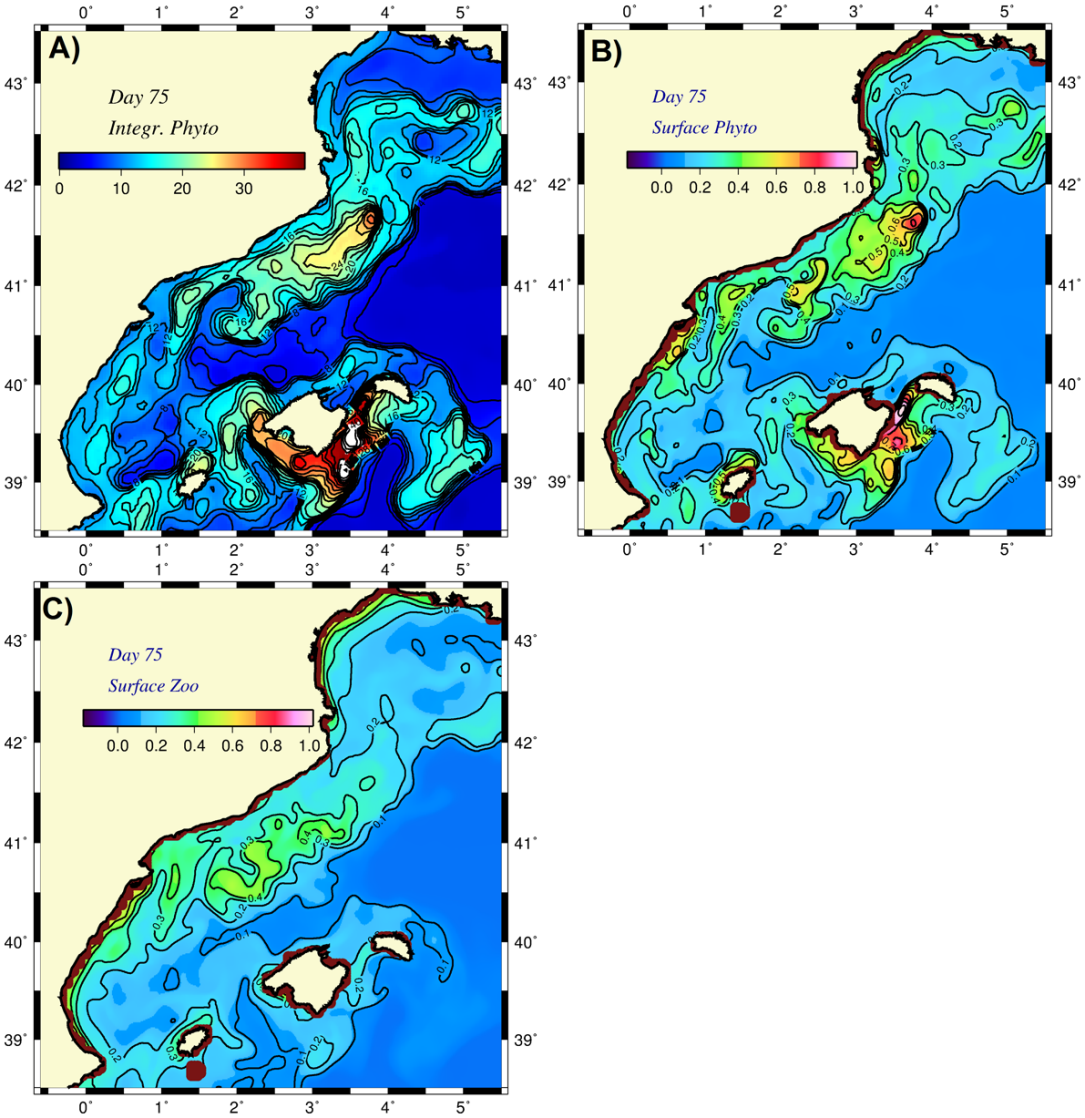
Distributions of (a) depth integrated (over 100 m) phytoplankton biomass, (b) surface phytoplankton biomass, (c) surface zooplankton biomass at day 75 for the diabatic simulation with buoyancy-induced cooling and higher zooplankton grazing rate.

The zooplankton grazing rate, one of the most critical biological parameters controlling changes in phytoplankton biomass, may vary regionally and seasonally within a large range and exert a large variability and uncertainty on the phytoplankton biomass structure. The previous buoyancy-induced cooling simulation that employed a grazing rate of 0.4 d^-1^ was repeated here with a value that was 50% larger value (0.6 d^-1^). In this case, the total phytoplankton biomass was reduced by almost three-fold from the typical 60 mmol m^-2^ to approximately 20 mmol m^-2^ (Fig 14a). This implies a change from the former autotroph-dominated system to a heterotroph-dominated one. The surface phytoplankton biomass values were approximately 0.5 mmol m^-3^ (Fig 14b). Surface zooplankton biomass was comparable to those of the phytoplankton at approximately 0.3–0.4 mmol m^-3^ (Fig 14c).

## Discussion and Conclusions

Numerous studies and field observations have noted the favorable roles of mesoscale features in quasi-geostrophic (i.e., weakly nonlinear) flow dynamics on the production and distribution of biological properties. However, there has been no quantitative support and analysis to date for the link between ageostrophic dynamics arising from the strongly nonlinear boundary current frontal instabilities and enhanced plankton production for marginal sea boundary current frontal systems. The present study attempted to fill this gap using a process-oriented biophysical model of the CB Sea. It specifically focused on winter conditions because they are the most productive period of the year with a more pronounced boundary current structure. However, the frontogenesis mechanism can operate during the entire year as long as the frontal boundary current is sufficiently nonlinear and unstable and primary production is not limited by other environmental factors. The mechanisms and model findings described here can also apply to other marginal seas with similar frontal boundary current systems.

In the absence of instabilities, a stable frontal boundary current could not support any appreciable biological production. In this case, the boundary current and the accompanying lower density coastal waters were primarily confined to the shelf-topographic slope zone and separated from the inert, more dense offshore waters by a well-defined stable front. Similarly, an unstable boundary current in the absence of cross-frontal density variations provided no biological production. In these cases, the front acted as an ecological barrier to cross-frontal exchanges. This structure was, however, altered in the case of frontal instabilities, depending on the degree of nonlinearity of the flow. The slope current that was confined to roughly 200–1400 m depths was accompanied on both sides by a shelf break and offshore fronts separating three different water masses: within the shelf (< 28.6 kg m^-3^ at the surface), the topographic slope zone (28.6–28.8 kg m^-3^), and beyond the continental margin topography (28.85–28.90 kg m^-3^). The slope current continued to flow further south and supported the Balearic current and mesoscale eddies within the Balearic interior basin.

In places where the boundary current and accompanying eddies were sufficiently nonlinear (measured by relative vorticity normalized to the planetary vorticity exceeding 0.5), they formed the frontogenesis-induced ageostrophic secondary circulation. These regions were characterized by upward vertical velocities of up to 80 m d^-1^ on the onshore (anticyclonic) side and comparable subduction on the offshore (cyclonic) side of the front. The upwelling motion was able to inject sufficiently high nutrient concentrations into the photic layer and promoted strong phytoplankton production. The resulting plankton biomass was then advected and distributed by the boundary current and its meanders, filaments and mesoscale eddies.

Atmospheric forcing modulated the ageostrophic frontal motion. Even for their relatively weak winter-mean values, down-front wind stress or heat flux (cooling) forcing intensified boundary current instabilities, upwelling velocities and thus phytoplankton production. Cooling strengthened nutrient entrainment from the subsurface within the frontal region where vertical velocities had transported nutrients near the base of the photic zone. However, the same vertical mixing in regions of weaker quasi-geostrophic upwelling velocities (on the order of a few meters per day mostly in offshore waters beyond the frontal zone) were unable to produce a comparable level of plankton production. Nevertheless, eddy pumping within some of the mesoscale eddies in the deep Balearic basin contributed to phytoplankton production, but the more predominant role for these eddies was to distribute nutrients and biota horizontally.

The ageostrophic frontal processes may also precondition the system for subsequent production events by accumulating nutrients at near-surface levels and recycled nutrients to be used under more favorable light and temperature conditions following the winter. This feature will be explored more thoroughly in future studies that incorporate the annual structure.

**Fig 15:**
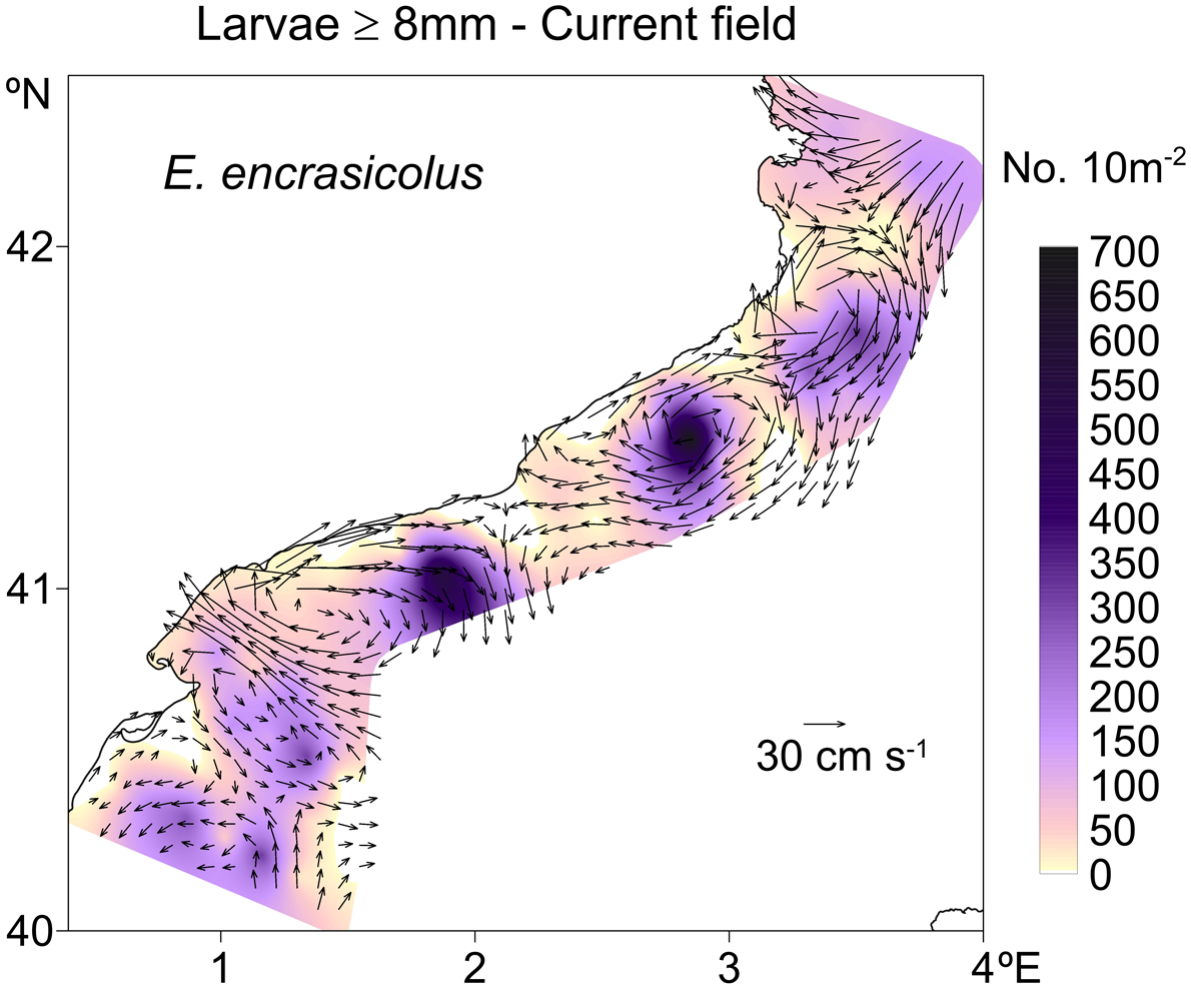
Distribution of anchovy larvae abundance and the geostrophically computed surface flow structure along the Catalan shelf-slope zone during June 2004 taken from Sabates et al. (2013).

Because the experiments performed in the present study were based on relatively idealized conditions and not designed to simulate any specific set of observations, we can only make a gross comparison between the model findings and available observations. For example, the anticyclonic vorticity regions between the Palamos and Blanes canyons, within the Merenguera canyon region and in the onshore meander near Barcelona agreed well with the observed features shown in Fig 15. This resemblance may point to the dominating role of the topographic steering mechanism in the path of the boundary current. The eddy-dominated flow structure near the Ibiza channel of the Balearic Sea was consistent observations [6, 48]. As expected, a more realistic atmospheric forcing may result in other forms of mesoscale-dominated flow structures within the Balearic Sea. Moreover, the spatial structure of the surface phytoplankton distribution (e.g., Fig 13b) closely resembled that of the surface chlorophyll concentration shown in Fig 2c.

The simulation with an increased zooplankton grazing rate described in the previous section agreed better with the observations. The phytoplankton biomass of approximately 0.5 mmol N m^-3^ corresponded to a chlorophyll concentration of approximately 1 mg m^-3^, which is consistent with observed concentrations provided by satellite ocean color data (e.g., Fig 2) and in situ measurements [5, 21]. This conversion derives from Chl = 12*P*RC:N/RC:Chl, with a carbon-to-nitrogen ratio of RC:N = 6.6, a carbon-to-chlorophyll ratio of RC:Chl = 50, and P representing the phytoplankton biomass in mmol m^-3^. Similarly, assuming that 1 mmol N m^-3^ zooplankton biomass amounts to 10 mg m^-3^ wet weight, the predicted levels of zooplankton biomass (e.g., Fig 13c) were comparable with their observed range of 3–5 mg ww m^-3^ reported by the long-term time series measurements near the Mallorca Island and within the Catalan Sea [21, 49].

The transport and retention characteristics of anchovy fish larvae along the Catalan coast demonstrates the roles of the meandering boundary current and ageostrophic dynamics [23, 24]. Anchovy larvae are transported southwards from their major spawning area in the Gulf of Lion by the shelf-slope frontal current and preferentially accumulate within coastal anticyclonic eddies (Fig 15) through convergence of the boundary current meandering anticyclonically towards the coast [23]. Conversely, during the offshore meandering phase of the boundary current, divergence causes dispersal of the larval population from the cyclonic eddies of the interior Catalan basin. The present study noted that the enhanced bottom-up resource supply near the peripheries of anticyclonic eddies on the onshore side of the frontal boundary current further supported the larval population that had aggregated within the coastal anticyclonic eddies. This contribution arises when the boundary current is sufficiently unstable to drive ageostrophic frontal processes at larvae retention sites.

Six successive surveys in 2003–2004 and previous observations identified a close connection between specific locations of high fishery activity and mesoscale flow characteristics within the Mallorca channel and its extension along the northern and southern regions of Mallorca Island [49, 50]. More recent observations from December of 2009 also documented an increase in mesozooplankton and fish biomass in regions of intense frontal activities [20]. Our model findings indeed provide quantitative support for this feature by showing enhanced phytoplankton biomass in anticyclonic eddies in this region. Next, modeling efforts should introduce a more complex and realistic food web structure to study the impacts of frontal dynamics on plankton diversity, succession and trophic interactions.
